# Performance comparison of four exome capture systems for deep sequencing

**DOI:** 10.1186/1471-2164-15-449

**Published:** 2014-06-09

**Authors:** Chandra Sekhar Reddy Chilamakuri, Susanne Lorenz, Mohammed-Amin Madoui, Daniel Vodák, Jinchang Sun, Eivind Hovig, Ola Myklebost, Leonardo A Meza-Zepeda

**Affiliations:** Department of Tumor Biology, Oslo University Hospital, Norwegian Radium Hospital, 0310 Oslo, Norway; Department of Medical Informatics, Oslo University Hospital, Norwegian Radium Hospital, Oslo, Norway; Norwegian Cancer Genomics Consortium, Oslo, Norway; Genomics Core Facility, Oslo University Hospital, Oslo, Norway; Department of Informatics, University of Oslo, Oslo, Norway

**Keywords:** Exome capture technology, Next-generation sequencing, Coverage efficiency, Enrichment efficiency, GC bias, Single nucleotide variant, Indel

## Abstract

**Background:**

Recent developments in deep (next-generation) sequencing technologies are significantly impacting medical research. The global analysis of protein coding regions in genomes of interest by whole exome sequencing is a widely used application. Many technologies for exome capture are commercially available; here we compare the performance of four of them: NimbleGen’s SeqCap EZ v3.0, Agilent’s SureSelect v4.0, Illumina’s TruSeq Exome, and Illumina’s Nextera Exome, all applied to the same human tumor DNA sample.

**Results:**

Each capture technology was evaluated for its coverage of different exome databases, target coverage efficiency, GC bias, sensitivity in single nucleotide variant detection, sensitivity in small indel detection, and technical reproducibility. In general, all technologies performed well; however, our data demonstrated small, but consistent differences between the four capture technologies. Illumina technologies cover more bases in coding and untranslated regions. Furthermore, whereas most of the technologies provide reduced coverage in regions with low or high GC content, the Nextera technology tends to bias towards target regions with high GC content.

**Conclusions:**

We show key differences in performance between the four technologies. Our data should help researchers who are planning exome sequencing to select appropriate exome capture technology for their particular application.

**Electronic supplementary material:**

The online version of this article (doi:10.1186/1471-2164-15-449) contains supplementary material, which is available to authorized users.

## Background

In general it remains prohibitively expensive to analyze whole genomes for population scale study, even though the cost of whole genome sequencing has fallen significantly [1]. As an alternative, the targeted resequencing of subsets of a genome is more feasible. The most widely used approach captures much of the entire protein coding region of a genome (the exome), which makes up about 1% of the human genome, and has become a routine technique in clinical and basic research [2–5]. Exome sequencing offers definite advantages over whole genome sequencing: it is significantly less expensive, more easily understood for functional interpretation, significantly faster to analyze, and an easy dataset to manage. Multiple technologies have surfaced for the enrichment of target regions of interest, as the demand for targeted resequencing has increased over time. Broadly, these technologies can be classified into two groups, chip-based exome capture versus solution-based exome capture. Chip-based exome capture was the first to be developed [6, 7], but required large amounts of input DNA, and was quickly replaced by more efficient solution-based capture systems. There are currently four major solution-based human exome capture systems available: Agilent’s SureSelect Human All Exon, NimbleGen’s SeqCap EZ Exome Library [8], Illumina’s TruSeq Exome Enrichment, and Illumina’s Nextera Exome Enrichment [9]. Exome capture involves the capture of protein coding regions by hybridization of genomic DNA to biotinylated oligonucleotide probes (baits). These technologies use biotinylated DNA or RNA baits complementary to targeted exons, which are hybridized to genomic fragment libraries. Magnetic streptavidin beads are used to selectively pull-down and enrich baits with bound targeted regions. The sample preparation methods are highly similar across the different technologies. The major differences between the technologies correspond to the choice of their respective target regions, bait lengths, bait density, molecules used for capture, and genome fragmentation method (Table 1).

**Table 1:** **Exome capture technology designs**

	NimbleGen	Agilent	Illumina TruSeq	Illumina Nextera
**Bait type**	DNA	RNA	DNA	DNA
**Bait length range (bp)**	NP	114-126	95	95
**Median bait length (bp)**	NP	119	95	95
**Number of baits**	NP	554,079	347,517	347,517
**Total bait length (Mb)**	NP	66.48	33.01	33.01
**Target length range (bp)**	59–742	114–21,747	2–37,917	2–37,917
**Median target length (bp)**	171	200	135	135
**Number of targets**	368,146	185,636	201071	201,071
**Total target length (Mb)**	64.19	51.18	62.08	62.08
**Fragmentation method**	Ultrasonication	Ultrasonication	Ultrasonication	Transposomes
**Automation**	++	++	++	+++
**Throughput**	+++	+++	+++	+++
**Flexibility**	Custom available	Custom available		Custom available
**Species**	Human, mouse, 3 plant species	Human, mouse, 14 other species custom	Human	Human
**Costs**	$$	$$	$	$

Some NimbleGen information was not provided, indicated by NP. Relative automation and throughput indicated by “+” symbols, higher number of symbols indicates easy to automate and higher throughput. Relative cost is indicated by “$” symbol, higher “$” symbols indicate the higher price.

Clark *et al*. compared three capture technologies and showed that NimbleGen technology required the least number of reads to sensitively detect small variants, whereas Agilent and Illumina technologies appeared to detect a higher total number of variants with additional reads [10]. In another study, Sulonen *et al*. compared NimbleGen and Agilent technologies, and showed that there were no major differences between the two technologies, except that NimbleGen showed greater efficiency in covering the exome with a minimum of 20x coverage [11]. Asan *et al*. compared NimbleGen Sequence Capture Array, NimbleGen SeqCap EZ, and Agilent SureSelect, and showed that all three technologies achieved a similar accuracy of genotype assignment and single nucleotide polymorphism (SNP) detection, and had similar levels of reproducibility and GC bias [12]. In another exome capture comparison study, Parla *et al*. showed that both NimbleGen SeqCap EZ Exome Library SR and Agilent SureSelect All Exon were similar to each other in performance, and able to capture most of the human exons targeted by their probe sets. However, they failed to cover a noteworthy percentage of the exons in the consensus coding sequence database (CCDS) [13].

During the past few years, substantial updates have been made to the different capture technologies, including new content and improved probe design. For instance, NimbleGen’s SeqCap EZ exome library v2.0 targets approximately 44 Mb of genome, where as their next version EZ exome library v3.0 targets 64.1 Mb. The new Illumina Nextera capture technology has to the best of our knowledge not been tested extensively vis-à-vis other technologies.

The lack of a clear consensus from previous studies, updates in three major capture technologies, and the important new Illumina Nextera capture technology, using an entirely different strategy, motivated us to perform a detailed comparative analysis before initiating a major exome sequencing project.

We, therefore, systematically compared four exome capture technologies, NimbleGen’s SeqCap EZ exome library v3.0, Agilent SureSelect Human all exon V4, Illumina TruSeq and Illumina Nextera, with respect to features such as design differences relative to coverage efficiency, GC bias, and variant discovery.

## Results

### Distinctive features of four exome capture technologies

**Figure 1:**
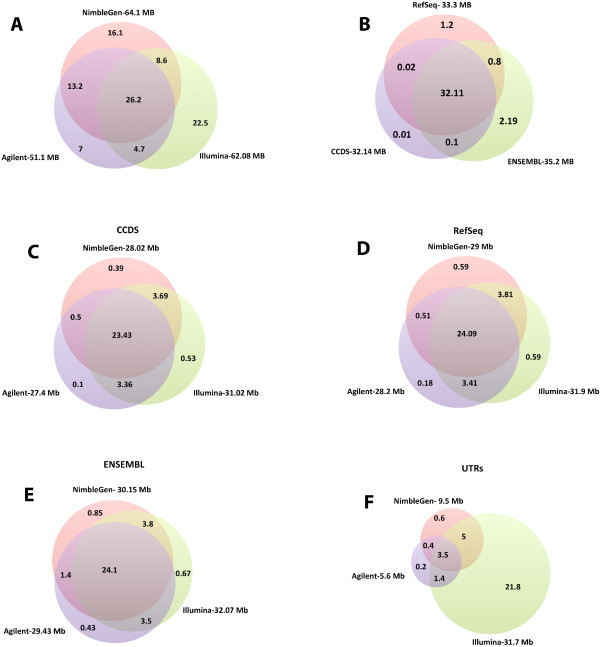
**Venn diagram showing the overlap between different features. A)** Overlap among Agilent, NimbleGen and Illumina capture targets. **B)** Overlap among RefSeq, CCDS, and ENSEMBL protein coding exon databases. Coverage of exome capture technology for **C)** CCDS coding exons, **D)** RefSeq coding exons, **E)** ENSEMBL coding exons, and **F)** RefSeq UTRs.

There are considerable differences between the four exome capture technologies, as shown in Table 1. Illumina TruSeq and Nextera technologies are identical in many characteristics, except that Nextera uses transposomes for fragmentation, whereas TruSeq fragments the DNA by ultrasonication. The Agilent technology uses RNA molecules as probes, whereas all the other technologies use DNA as probe molecules. NimbleGen presents the highest number of probes, being the only technology with an overlapping probe design, thus giving it the highest probe density technology of the four. Agilent probes are non-overlapping, but lie directly adjacent to one another. On the other hand, the Illumina technologies, use a gapped probe approach. The technologies also differ in the regions they target, and in the total number of bases targeted. For instance, NimbleGen targets 64.1 Mb, Agilent targets 51.1 Mb, and TruSeq and Nextera targets 62.08 Mb of human genome.Interestingly, only 26.2 Mb of the total targeted bases are common among all exome capture technologies (Figure 1A). Of the four, NimbleGen and Agilent technologies have the most in common, sharing almost 40 Mb of targeted sequences. Illumina has 22.5 million unique target bases, followed by NimbleGen with 16.1 million bases, and Agilent with 7 million unique bases.

Many different RNA databases are available, such as RefSeq [14] and Ensembl [15], which differ in the number of non-coding RNAs and total number of exons reported, as well as the start and end coordinates of exons. Significant portions of the sequences are common among the different databases (Figure 1B). CCDS contains protein-coding sequences with high quality annotations [16]. RefSeq and CCDS share a greater proportion of bases with each other, whereas Ensembl possesses more unique bases (2.19 million) than the other two databases. We investigated the coverage of RefSeq (coding and UTR), Ensembl (coding) and CCDS (coding).Illumina covers a greater portion of coding exon bases across all the databases, followed by NimbleGen and Agilent (Figure 1C–E). There are 32.11 Mb common across the three databases, but only about 24 Mb are covered by all four technologies. The majority of Illumina-specific bases (22.5 Mb) target untranslated regions (UTRs) (Figure 1F), whereas NimbleGen and Agilent target UTRs at 9.5 Mb and 5.6 Mb, respectively.

### Sequencing, sequence alignment, and read filtering

**Figure 2:**
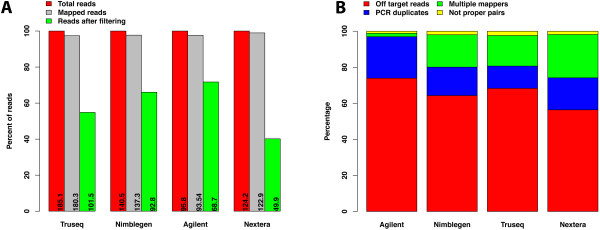
**Read statistics. A)** Bar plot showing percent of initial reads, mapped reads and reads left after filtering for four different technologies; each bar shows the number of reads in millions. **B)** Stacked bar plot showing subgroups of filtered reads.

To evaluate each technology, two independent exome libraries derived from the tumor tissue of an osteosarcoma sample were sequenced twice (technical replicates). The exome library for each technology was prepared according to each supplier’s recommended protocol. On average, 136.8 million reads were generated for each technology, varying between 95.8 and 185.1 million reads. There were also differences in sequencing and alignment rates between the different technologies. The read alignment rate varied among technologies: 97.4% for TruSeq, 97.7% for NimbleGen, 97.6% Agilent, and 98.95% for Nextera (Figure 2A). Mapped reads from each library were further filtered for duplicates, multiple mappers, improper pairs, and off-target reads. Large variation was observed for the percentage of pass-filter mapped reads, with Agilent being the highest at 71.7% retained reads, NimbleGen next at 66.0%, TruSeq at 54.8%, and Nextera at 40.1% (Figure 2A). We further examined the number of reads filtered out in each of the four steps (Figure 2B). For all the technologies, the greatest number of reads lost was due to the number of reads mapped to non-targeted regions (off-target reads). Agilent showed a slightly higher percentage of off-target reads and the fewest reads mapping to multiple sites.

### Target coverage efficiency differs among four technologies

**Figure 3:**
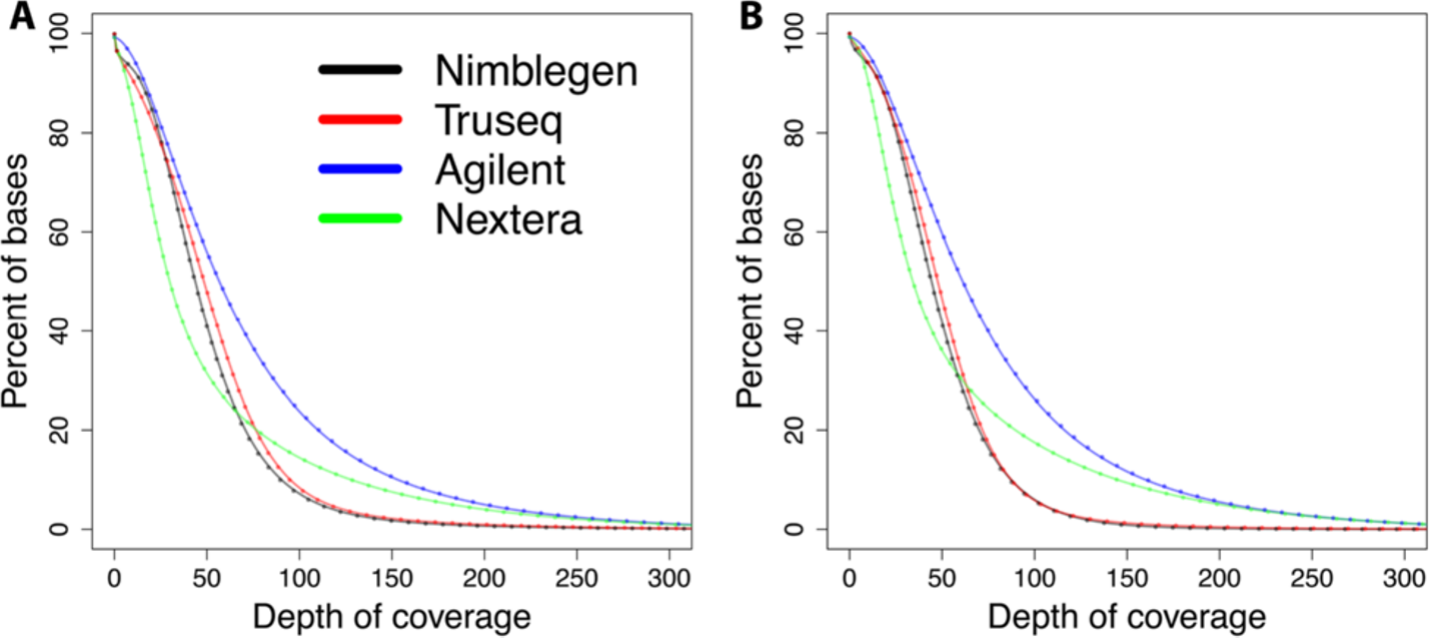
**Coverage efficiency comparison by technology.** Coverage efficiency defined as the percent of the total targeted bases covered at particular depths. **A)** Coverage efficiency for intended targeted bases for each technology. **B)** Coverage efficiency for bases, which are shared, by all four technologies (26.2 MB). Smooth line indicates replicate 1, and dotted line indicates replicate 2.

**Figure 4:**
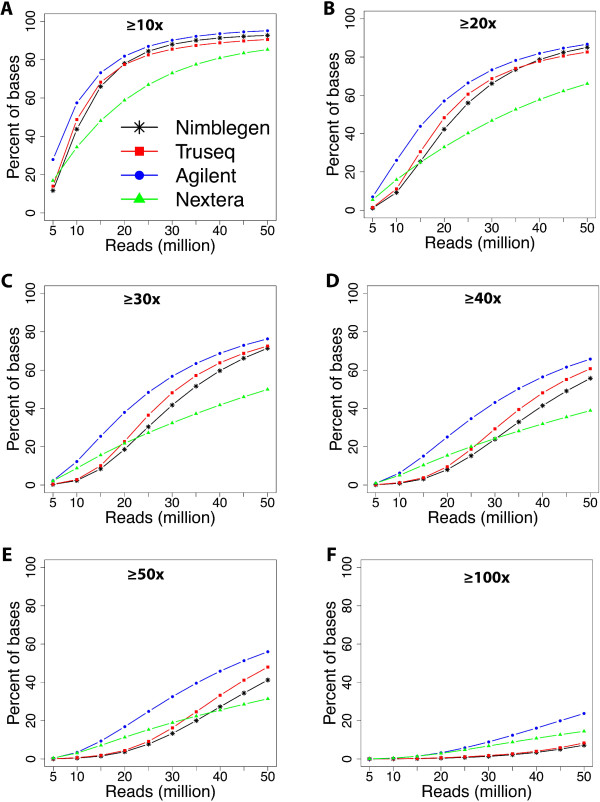
**Coverage efficiency as a function of number of reads.** The percent of targeted bases covered at **A)** ≥10x, **B)** ≥20x, **C)** ≥30x, **D)** ≥40x, **E)** ≥50x, and **F)** ≥100x depths.

We used the methods described by Clark et al. [10] to investigate target coverage efficiency. We evaluated coverage efficiency by calculating base coverage over 1) all intended target bases, 2) common bases among the four technologies, 3) Ensembl exons, 4) RefSeq exons, and 5) CCDS exons, using 50 million randomly chosen reads for each technology. Target coordinates were downloaded from the supplier’s websites. It is worthwhile to note that TruSeq and Nextera, both supplied by Illumina, use the same capture baits. At this level of reads, the fractions of targets covered at least once varied somewhat, the Agilent technology captured 99.8%, the Nextera technology captured 98.2%, the TruSeq captured 96.9%, and the NimbleGen captured 96.5% of the intended targets (Figure 3A). The 1× coverage number provides the fraction of the target that can potentially be covered by the respective designs. Not surprisingly, all the technologies give high coverage of their respective target regions, with the Agilent technology giving highest coverage (99.8%). The number of intended target bases varies considerably, as the Agilent technology targets 51.1 Mb, NimbleGen 64.1 Mb, and Illumina 62.08 Mb (Figure 1A), sharing only 26.2 million bases between technologies. When measured at 1× coverage on the common bases (26.2 Mb), we observed a similar trend, where the Agilent technology covers the highest number of bases, with 99.8%, followed by Nextera with 99.5%, TruSeq with 98.8%, and NimbleGen with 98% (Figure 3B and Additional file 1: Figure S1). We found no major difference in coverage efficiency between two technical replicates, indicating that all four technologies give high technical reproducibility.We next evaluated coverage efficiency as a function of sequencing depth. We randomly selected filtered reads in 5 million read increments from 5 million to 50 million. The fraction of the intended target bases, covered at depths of at least 10×, 20×, 30×, 40×, 50× and 100×, was determined (Figure 4). The Agilent technology covered a higher percent of its target bases at all read counts and depth cut-offs compared with the other three technologies. For all the technologies, 25 million reads were sufficient to cover about 80% of target bases with at least 10× depth, with the exception of the Nextera technology, which covered only about 60% of target bases with the same number of reads (Figure 4A). When using 45 million reads with all the technologies, more than 80% of target bases were covered with ≥20× coverage, but the Nextera technology covered only 58% of the bases at the same depth (Figure 4B). For all the read counts, Agilent and Nextera covered more bases with ≥100× coverage than other two technologies, but showed a considerable difference in coverage (Figure 4F).

### Influence of GC content on coverage

**Figure 5:**
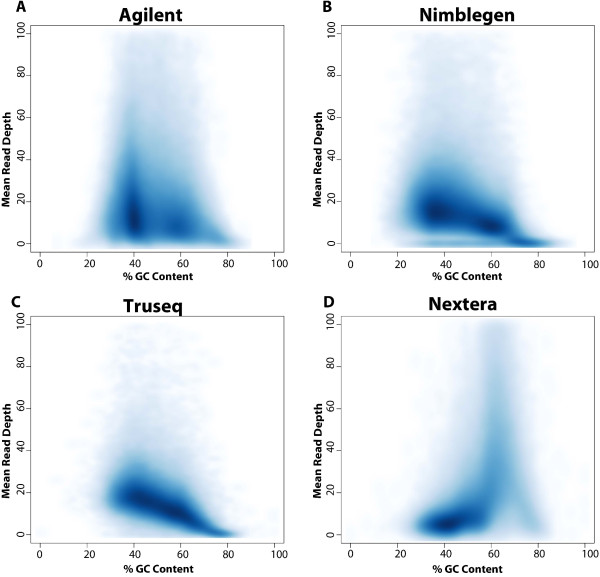
**Density plots showing GC content against normalized mean read depth for A) Agilent, B) NimbleGen, C) TruSeq, and D) Nextera technologies.**

Base composition has been shown to bias sequencing efficiency, thus coverage may be low for sequences with high GC or AT content [17]. There are two primary explanations for this bias: 1) a polymerase chain reaction (PCR) amplification bias, where high or low GC content reduces the efficiency of PCR amplification [18]; and 2) a reduced efficiency of capture probe hybridization to sequences with high or low GC content [19]. Whereas the former bias is inherent of the sequences to be amplified, the latter is a property of the capture probes, and may to some extent be compensated by probe design. To study the GC bias effect, we utilized density plots as described by Clark et al. [10], where we plotted GC content against the normalized mean read depth (Figure 5 and Additional file 2: Figure S2). All four technologies showed bias against very low (<30%) and very high (>70%) GC content. All the technologies, except Nextera, demonstrated a sharp fall in read depth for GC contents of 60% or higher. Nextera gave increased coverage for sequences with higher GC content, owing to the preference of the transposon technology used [20]. All the technologies gave poor coverage for sequences with less than 25% GC content.

### Ability to detect SNVs

**Figure 6:**
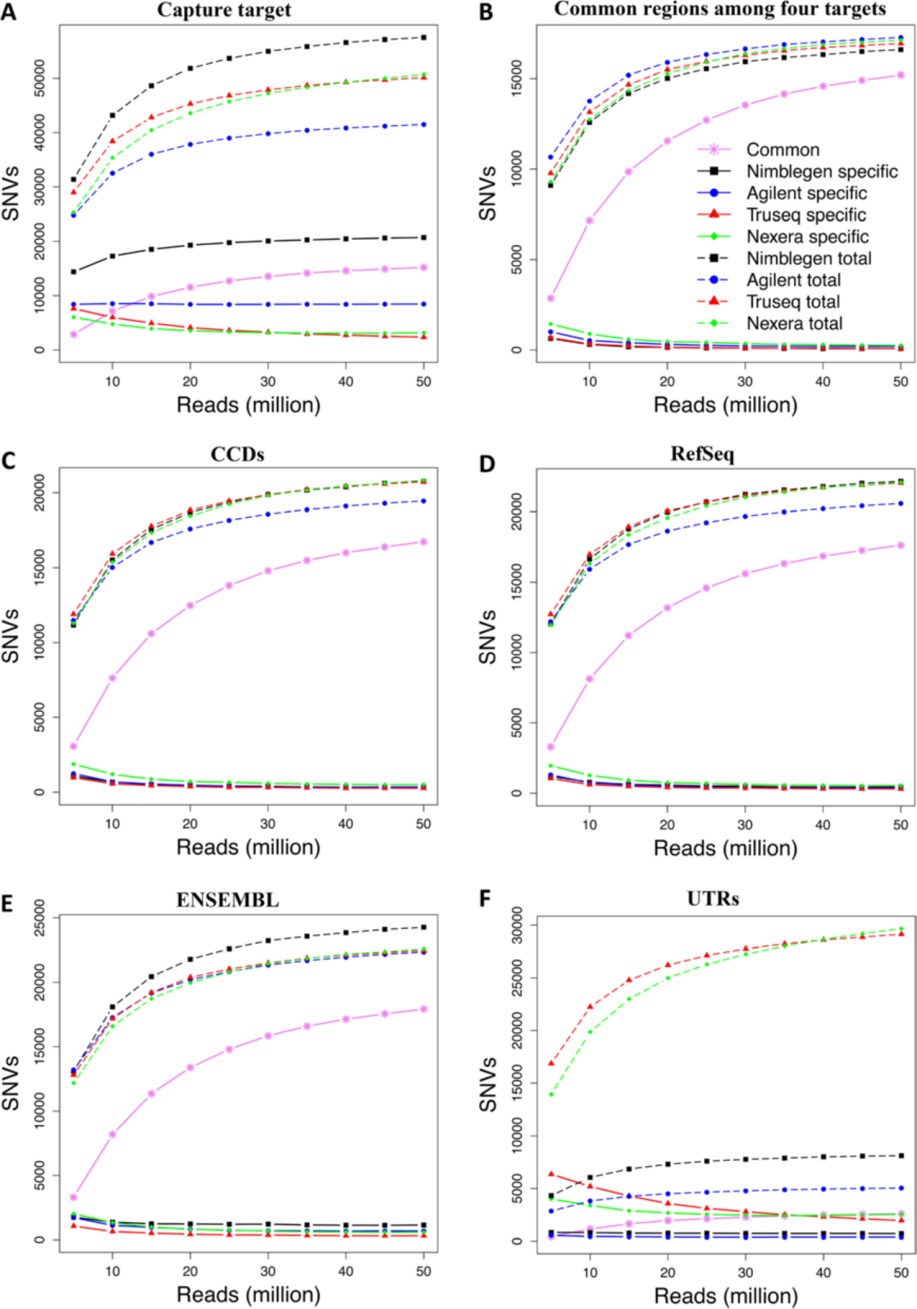
**SNV detection by technology as a function of increasing read counts on A) intended target region, B) regions common among technologies, C) CCDS exons, D) RefSeq exons, E) Ensembl exons, and F) UTRs.** Solid-lines indicate technology specific SNVs, dashed-lines indicate total number of SNVs, and solid pink lines indicate the SNVs common between the four technologies.

An important goal of exome resequencing is to identify sequence variants. Therefore, we systematically compared the efficiency of exome capture for allele detection among the four technologies. We used UnifiedGenotyper, implemented in the GATK package [21], to investigate the relationship between read counts and total single nucleotide variants (SNVs) detected within different intervals. As read counts increased, the number of SNVs identified in their target regions increased initially, and became saturated at approximately 20 million reads (Figure 6A). Very few additional SNVs were identified beyond 20 million reads. When considering the SNVs identified on their respective target regions, there is a clear correlation between the total number of SNVs detected and the number of bases targeted; NimbleGen detected the highest number of SNVs followed by TruSeq, Nextera, and Agilent (Figure 6A and Additional file 3: Figure S3A). A different trend was clear in the 26 Mb region shared by all four technologies, where Agilent detected the highest number of SNVs, followed by Truseq, Nextera, and NimbleGen (Figure 6B and Additional file 3: Figure S3B). The majority of newly detected SNVs were common.We also investigated SNV detection in the regions covered by the CCDS (Figure 6C), RefSeq (Figure 6D), and Ensembl (Figure 6E) exome databases. The Illumina technologies, TruSeq and Nextera, and NimbleGen detected similar number of SNVs in CCDS and RefSeq. However in Ensembl regions, NimbleGen detected the highest number of SNVs. As expected, Illumina technologies detected a much larger number of SNVs in UTRs. Illumina technologies also covered the highest number of bases in the UTRs, followed by NimbleGen and Agilent (Figure 1F). Interestingly, at low read counts, more SNVs were detected by TruSeq, but at 40 million read counts, Nextera surpassed TruSeq.

We also investigated whether capture technologies showed bias in substitution detection, but none of the technologies showed bias towards specific nucleotide substitutions (Additional file 4: Figure S4 and Additional file 5: Figure S5). Transitions were expected to occur twice as frequently as transversions. The transition-transversion (ts/tv) ratio is a metric for assessing the specificity of new SNP calls. We assessed the ts/tv ratio on their respective target regions (including non-exonic segments), and it ranged from 2.215 in Nextera to 2.257 in Agilent (Additional file 4: Figure S4). Previous studies have shown ts/tv ratios of ≈ 2.0–2.1 for whole genome datasets [22]. The Nextera and TruSeq technologies showed very similar ts/tv ratios, caused most likely by their identical target regions. Also, Agilent and NimbleGen had very similar ts/tv ratios. The difference in ts/tv ratios between Illumina technologies (TruSeq and Nextera) and non-Illumina technologies (Agilent and NimbleGen) may be because Illumina technologies target a significantly higher number of UTRs than the other technologies. We also determined the ts/tv ratio in CCDS coding exons (Additional file 5: Figure S5). The ts/tv ratio on CCDS ranges from 3.054 in Nextera to 3.109 in NimbleGen. It has been previously shown that the ts/tv ratio is ≈ 3.0–3.3 for exonic variation [23].

### Detection of insertions and deletions

**Figure 7:**
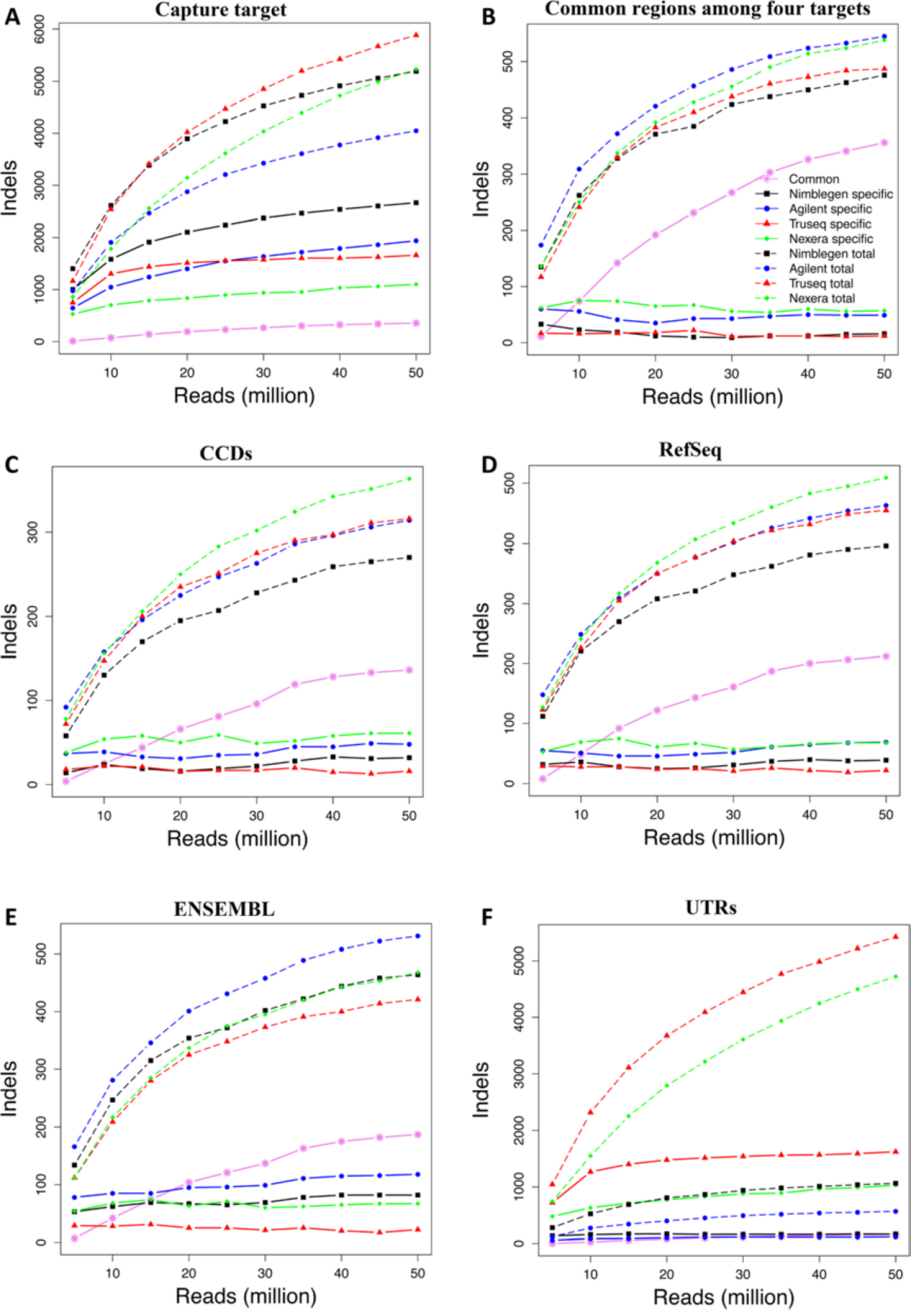
**Indels detection by technology as a function of increasing read counts on A) intended target region, B) regions common among the technologies, C) CCDS exons, D) RefSeq exons, E) Ensembl exons, and F) UTRs.** Solid-lines indicate technology specific SNVs, dashed-lines indicate total number of SNVs, and solid pink lines indicate the SNVs common between four technologies.

Small insertions and deletions (indels) were called using the UnifiedGenotyper algorithm implemented in the GATK package [21]. Indel size ranged from −40 to +37 bases in Agilent, −61 to +37 bases in NimbleGen, −66 to +52 bases in TruSeq, and −66 to +90 bases in Nextera. Most indels were single bases, and more than 90% of the indels were less than seven bases long; this pattern was observed for all four technologies (Additional file 6: Figure S6A). At low read counts, TruSeq and NimbleGen detected a higher number of indels, followed by Nextera and Agilent (Figure 7A). At 15 million read counts, TruSeq surpassed NimbleGen, and at 20 million reads, Nextera surpassed Agilent (Figure 7A). Interestingly, at 50 million reads, Nextera surpassed NimbleGen (Figure 7A). At all the read counts, a disturbing fact was that very few indels were common across the four technologies, especially on CCDS, Ensembl and RefSeq regions.Figure 7B shows a head-to-head comparison of indel detection in the regions covered by all four technologies. At all read counts, Agilent detected the highest number of indels. At lower read counts, NimbleGen detected more indels than TruSeq and Nextera; at 15 million reads, both Nextera and TruSeq surpassed NimbleGen. Only about 50% of indels were common among four technologies.

Indel detection in the regions covered by exome databases was also studied (Figure 7C–E). The number of indels detected in exons was significantly lower, than indels detected on the respective technology target regions and UTRs. We observed more indels of three or six bases (Additional file 6: Figure S6B), probably due to the negative selection of sizes not equal to multiples of three bases in coding sequences because they cause deleterious frame shift mutations.

When compared between replicates, both SNVs (Additional file 7: Figure S7 and Additional file 8: Figure S8) and indels (Additional file 9: Figure S9), showed similar trends in detecting total number of variants and showed very high overlap in newly detected variants.

## Discussion

Continuous advancement in sequencing technologies increases the throughput of DNA sequencing, while at the same time contributes sharply to decreasing its cost. Although sequencing costs have fallen, whole genome sequencing is still quite expensive, and data interpretation remains challenging. Therefore, whole genome sequencing is not the most appropriate choice for all investigations. The ability to target certain regions of the genome, such as protein and or RNA-coding exons, is an attractive alternative for many experiments. In recent times, target enrichment by hybridization technologies has demonstrated rapid progress in development and usage by the research and diagnostic community.

We present a comparative study of four whole exome capture technologies from three manufacturers, designed to reveal important performance aspects of the technologies. To address this, we studied six parameters for each technology: the portion of target bases representing different exome databases, target coverage efficiency, GC bias, sensitivity in SNV detection, sensitivity in small indel detection, and reproducibility.Although all four exome capture technologies show very high target enrichment efficiency and cover large portions of the exome, only a small portion of the CCDS exome is uniquely covered by each technology (Figure 1C). Therefore, a researcher who is planning exome sequencing should assess which technology best covers the regions of interest to the investigation. Agilent targets the smallest part of the genome with 51.1 Mb, followed by Illumina technologies with 62.08 Mb, and NimbleGen with 64.1 Mb. There are 26.2 Mb of the human genome shared by all four technologies; the majority of which falls in CCDS exonic regions. Illumina not only encompasses far more UTRs, but also shows a higher coverage of RefSeq, CCDS, and Ensembl exome databases, followed by NimbleGen and Agilent.

Target coverage efficiency differs between the four technologies. Using pass-filter reads, Agilent shows higher coverage efficiency than the other technologies, which may be partially explained by the smaller targeted region (51.1 Mb) compared with 64.1 Mb and 62.08 Mb for NimbleGen and Illumina respectively. Among the Illumina technologies, TruSeq gave a more uniform coverage than Nextera, but both had inferior efficiency compared with Agilent. Agilent gives the highest percentage of usable reads (pass-filter reads) (71.7%), closely followed by NimbleGen.

Regardless of high or low target region GC content, there was a negative correlation between sequencing coverage and extreme GC content. Preference for transposon targets with high GC content can help explain non-uniform coverage for the Nextera technology.

Most researchers aiming for exome sequencing, especially in the medical sciences, focus on protein-coding regions. Therefore, the ability to identify SNVs and indels in coding regions is critical to many applications. NimbleGen captures the highest number of SNVs, followed by Illumina technologies and Agilent, when the total number of SNVs detected are correlated with technology target size. However, the number of bases sequenced also has cost and capacity considerations. Our results suggest that Illumina technologies detect a higher number of SNVs over the other technologies with regard to SNV detection against the CCDS and RefSeq exomes, owing to a higher coverage of these regions, but Agilent was better at detecting indels. We also observed that Nextera shows a clear edge over other technologies in the CCDS and RefSeq exomes, because it covers a larger fraction of these sequences.

We did not observe significant differences in technical reproducibility between the four technologies. However, we could, by comparing performance between replicates to the differences observed above, conclude that although some differences in SNV and indel detection were due to random experimental error, the major effect appears to be due to technological biases.

Since the comparison is based on a tumor sample, which may contains genomic aberrations that could differentially affect the performance of each technology, we investigated the coverage differences in COSMIC cancer genes. No significant deviation in coverage was observed when compared with global coverage (Figure 3 and Additional file 10: Figure S10).

Another important consideration is exome capture technologies evolve rapidly. For instance, Agilent recently released their next version of exome capture SureSelect Human All Exon V5. Although these versions do differ with regard to the genomic regions they target, about 84% of target region bases overlap. Illumina also has a new version, with a smaller targeted panel, just for exons. It is called Nextera Rapid Capture Exome (37 Mb), while the larger panel version is now named Nextera Expanded Exome (62 Mb). Illumina has also improved the Nextera protocol, with the Nextera Rapid kit; this improvement may reduce the GC bias observed here.

In total, our data suggest that all four technologies offer comparable performance. Other factors, such as the DNA content of the targeted regions, the amount of input DNA required, the extent of automation in library construction, and the cost of reagents to reach a certain depth of coverage, need to be considered before selecting the exome capture technology most appropriate for your particular application.

Readers should keep in mind that this study is based on one biological sample with two replicates. The observed technical reproducibility is very high and variability may be higher when two biological replicates are compared.

## Conclusions

We systematically evaluated the performance of four whole exome capture technologies, and show that all the exome capture technologies perform well, but do exhibit consistent differences. Illumina covers a greater portion of coding exon bases across all the databases, followed by NimbleGen and Agilent. All the technologies give high coverage of their respective target regions, with the Agilent technology giving highest coverage (99.8%) followed by Nextera (98.2%), Truseq (96.9%), and NimbleGen (96.5%) of the intended targets. Nextera shows a sharp increase in read depth for GC content of 60% or higher compared other technologies. In common regions covered by all four technologies, Agilent detects slightly higher number of SNVs, followed by Nextera, TruSeq and Nimblegen. At all the read counts very few indels were common across the four technologies. All technologies give high technical reproducibility. One major limitation is that none of the capture technologies are able to cover all of the exons of the CCDS, RefSeq or Ensembl databases. Our study should help researchers who are planning exome sequencing experiments select the most appropriate technology for their study, without having to perform expensive and time-consuming comparisons.

## Methods

### Sample collection and library preparation

One human osteosarcoma was selected from a tumor collection at the Department of Tumor Biology at the Norwegian Radium Hospital. The tumor was collected immediately after surgery after written informed consent, cut into small pieces, frozen in liquid nitrogen and stored at −70°C until use.

High quality genomic DNA was isolated using the Promega Wizard Genomic DNA Purification Kit. One μg of genomic DNA was used to produce each exome captured sequencing library for four different technologies: NimbleGen SeqCap EZ v3.0, Agilent SureSelect XT2 Human All Exome v4.0, Illumina TruSeq Exome Enrichment kit and Illumina Nextera Exome Enrichment kit. The exome captured library preparation from the last three technologies was done following the manufacturers’ protocols applying pre-capture multiplexing. The protocol for NimbleGen SeqCap EZ was adapted from the company’s application note (http://www.nimblegen.com/products/lit/NimbleGen_SeqCap_EZ_SR_Pre-Captured_Multiplexing.pdf). The exome captured sequencing libraries were quality-controlled using an Agilent 2100 Bioanalyzer, and quantified using the Agilent QPCR NGS Library Quantification Kit (illumine GA) prior to cluster generation on an Illumina cBot.

### Datasets

The human reference genome (hg19), RefSeq, CCDS, and Ensembl databases were downloaded from the UCSC genome table browser (http://genome.ucsc.edu/).

Because of Norwegian legal regulations, the ethical approval for this study and the consent signed by the patient, we are not able to deposit our dataset in a public repository. We will provide access to the data if requested.

### Sequencing and bioinformatics data analysis

**Figure 8:**
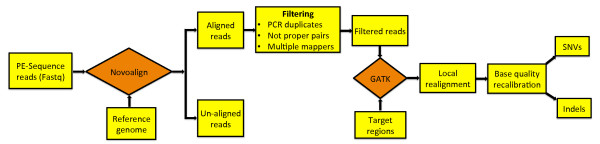
**Overview of the computational pipeline.**

Sequencing of each exome capture library was done at the Oslo University Hospital Genomics Core Facility, using an Illumina HiSeq 2000 machine, as pair-end 100-bp reads, following the manufacturer’s protocols using TruSeq SBS v3. We developed an in-house pipeline for analysis, which integrates several existing programs (Figure 8).

Briefly, initial FASTQ files were subjected to quality control with the FastQC tool (http://www.bioinformatics.babraham.ac.uk/projects/fastqc/). Raw reads from each capture library were aligned to the human reference genome (hg19) with Novoalign (http://novocraft.com/), using default parameters. If more than one pair (PE sequencing) had identical start and end coordinates, they were considered PCR duplicates and were removed using in-house scripts. Filtered read counts were normalized to 50 M reads between all four exome capture sequencing experiments by randomly selecting 50 M reads from each filtered read set. These randomly selected sets were further used to select 5–50 M reads, using an increment of 5 M reads.

SNVs and indels were called with GATK [21]. The GATK pipeline was independently run on each data set. We followed the procedure recommended by the GATK documentation. Reads around indels were realigned. To remove systematic biases in quality scores, base quality score recalibration was done. The UnifiedGenotyper algorithm was run using a *stand_emit_conf* of 10.0 and *stand_call_conf* of 30.0. All variants with a Phred-based quality score <30.0 were called low quality and ignored.

## Electronic supplementary material

Additional file 1: Figure S1: Coverage efficiency shown as a bar plot for different depths for replicate 1. (PNG 798 KB)

Additional file 2: Figure S2: Coverage efficiency on high (>70% GC) and low GC (<30% GC) regions, as a function of number of reads. The percent of targeted bases covered at **A)** ≥10x **B)** ≥20x **C)** ≥30x **D)** ≥40x **E)** ≥50x and **F)** ≥100x depths. (PNG 2 MB)

Additional file 3: Figure S3: Comparison of SNVs detection by each technology at 50 million reads. **A)** SNVs detected on intended target regions, and **B)** SNVs detected on regions shared by all four technologies. (PNG 861 KB)

Additional file 4: Figure S4: Mutation spectra by technology on intended target regions. Bar plots showing relative mutation frequency of different types of mutations for **A)** Agilent, **B)** NimbleGen, **C)** TruSeq, and **D)** Nextera technologies. Transition/Transversion (ts/tv) ratio indicated. (PNG 686 KB)

Additional file 5: Figure S5: Mutation spectra from CCDS exonic regions by technology. Bar plots show the relative mutation frequency of different types of mutations for **A)** Agilent, **B)** NimbleGen, **C)** TruSeq, and **D)** Nextera technologies. Transition/Transversion (ts/tv) ratio indicated. (PNG 688 KB)

Additional file 6: Figure S6: Indel size distribution by technology. **A)** Size distribution of all the indels that fall within the technology target regions. **B)** Size distribution of indels in CCDS coding exons. (PNG 2 MB)

Additional file 7: Figure S7: Comparison between two technical replicates in detecting SNVs, for **A)** Agilent, **B)** NimbleGen, **C)** TruSeq, and **D)** Nextera technologies. Smooth lines indicate SNVs detected on respective target regions, and dotted lines indicate SNVs detected on the target regions shared by all four technologies. Each figure shows the total number of SNVs detected by each replicate, common SNVs between replicates, and technology specific SNVs. (PNG 1 MB)

Additional file 8: Figure S8: Comparison of SNVs detection by each technology at 50 million reads on regions shared by all four technologies between two replicates for **A)** Agilent, **B)** NimbleGen, **C)** TruSeq, and **D)** Nextera technologies. (PNG 585 KB)

Additional file 9: Figure S9: Comparison between two technical replicates in detecting indels, for **A)** Agilent, **B)** NimbleGen, **C)** TruSeq, and D) Nextera technologies. Smooth lines indicate indels detected on intended target regions, and dotted lines indicate indels detected on the target regions shared by all four technologies. Each figure shows the total number of indels detected by each replicate, common indels between the replicates, and technology specific indels. (PNG 1 MB)

Additional file 10: Figure S10: Coverage efficiency comparison by technology on cancer genes. The smooth line indicates replicate 1 and the dotted line indicates replicate 2. (PNG 703 KB)

## Abbreviations

Bp: Base pair
CCDS: Consensus coding sequence
GATK: Genome analysis toolkit
indel: Insertion-deletion
miRNA: microRNA
SNP: Single nucleotide polymorphism
SNV: Single nucleotide variant
Mb: Megabase
ts: Transition
tv: Transversion
pcr: Polymerase chain reaction
UTRs: Untranslated regions.
