# Supplementary figures and images for: Performance comparison of four exome capture systems for deep sequencing
Source: BMC Genomics. 2014 Jun 9;15(1):449. doi: 10.1186/1471-2164-15-449 (PMC4092227; doi:10.1186/1471-2164-15-449)

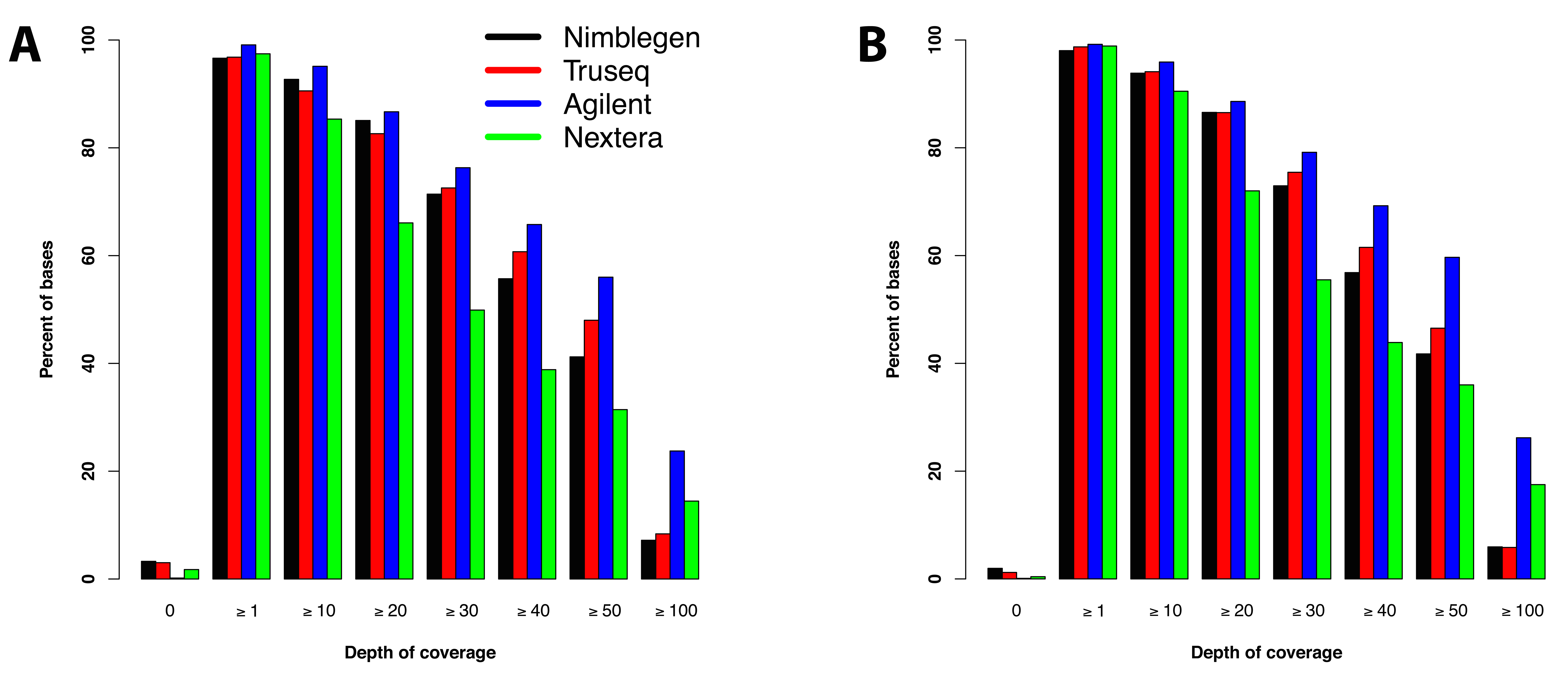

Supplement: Supplementary file 1 — Additional file 1: Figure S1: Coverage efficiency shown as a bar plot for different depths for replicate 1. (PNG 798 KB) [file 12864_2013_6212_MOESM1_ESM.png]

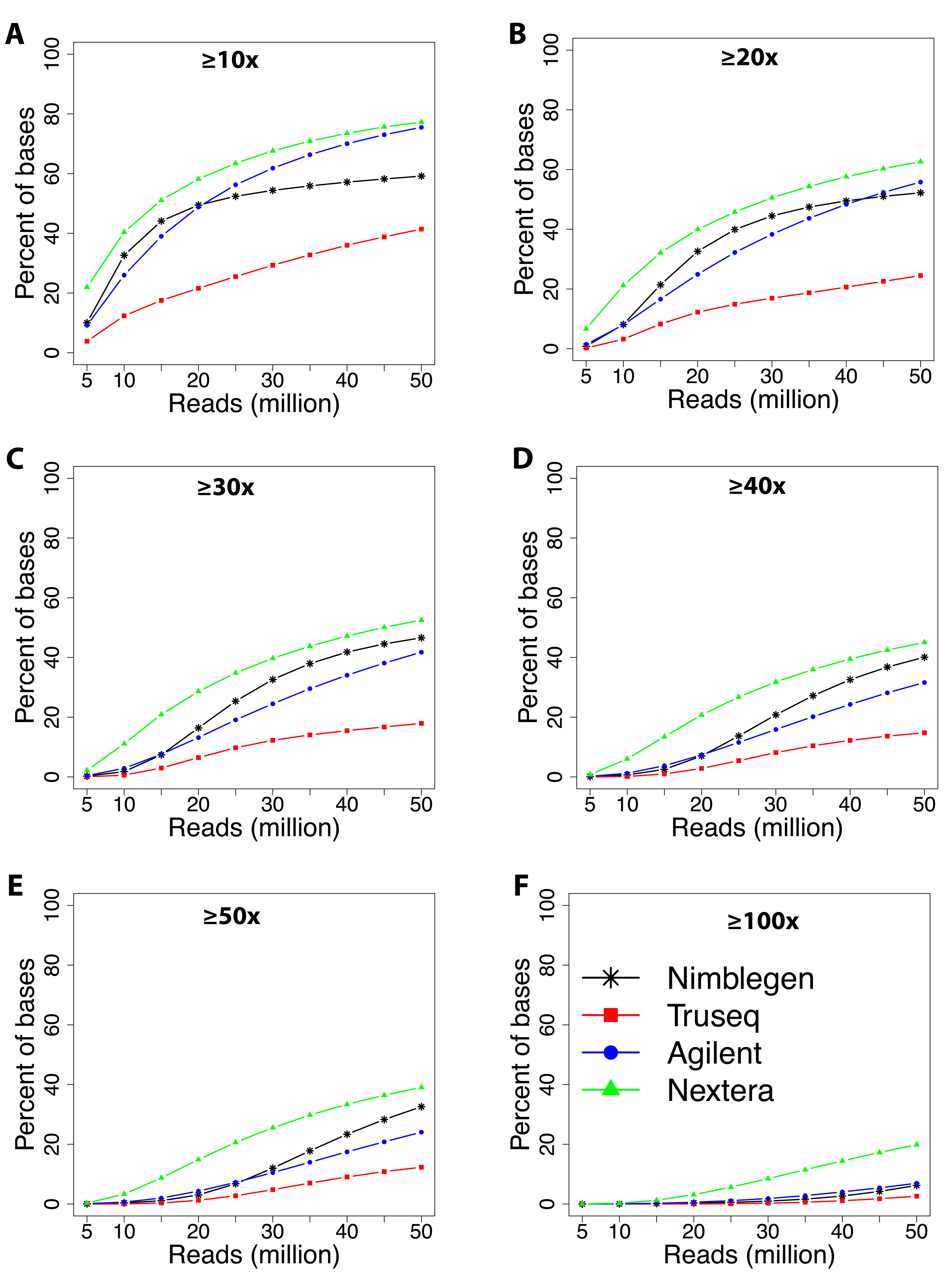

Supplement: Supplementary file 2 — Additional file 2: Figure S2: Coverage efficiency on high (>70% GC) and low GC (<30% GC) regions, as a function of number of reads. The percent of targeted bases covered at A) ≥10x B) ≥20x C) ≥30x D) ≥40x E) ≥50x and F) ≥100x depths. (PNG 2 MB) [file 12864_2013_6212_MOESM2_ESM.png]

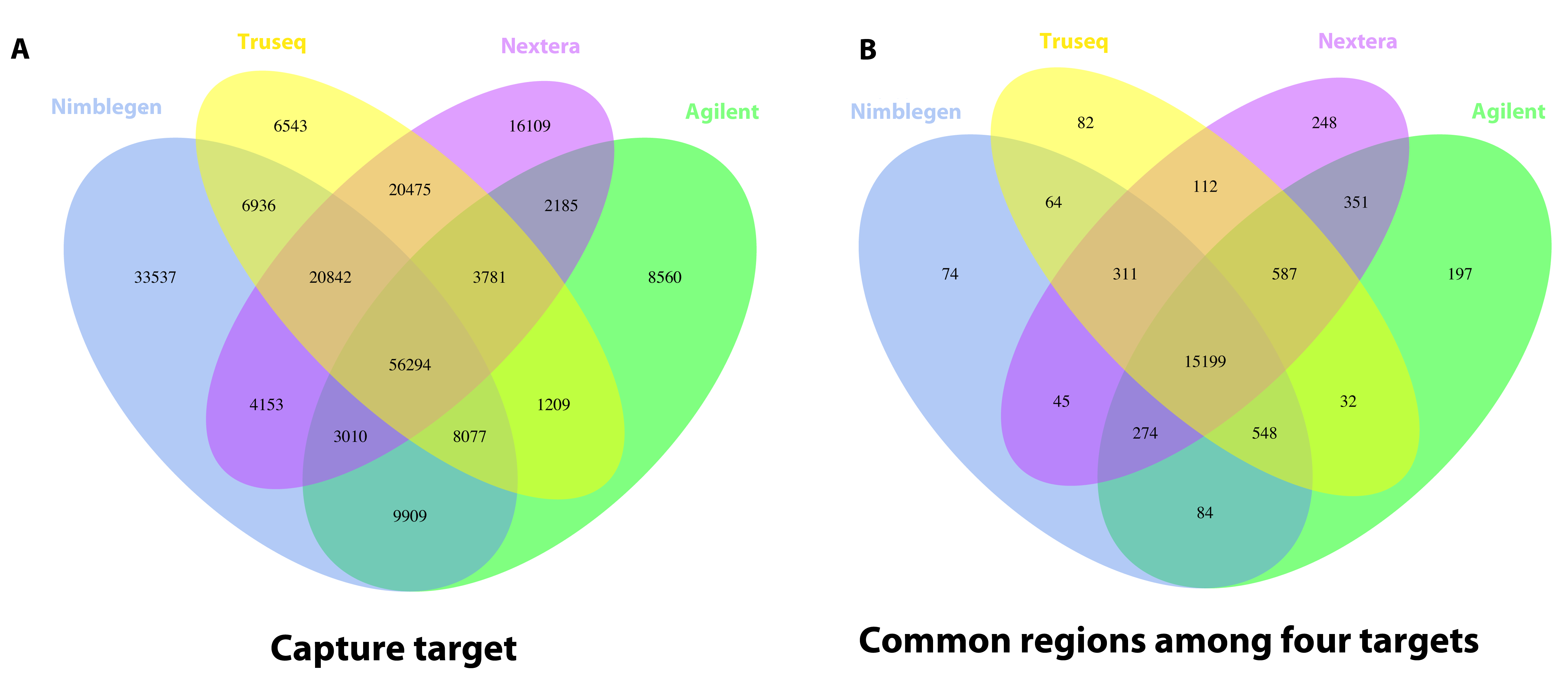

Supplement: Supplementary file 3 — Additional file 3: Figure S3: Comparison of SNVs detection by each technology at 50 million reads. A) SNVs detected on intended target regions, and B) SNVs detected on regions shared by all four technologies. (PNG 861 KB) [file 12864_2013_6212_MOESM3_ESM.png]

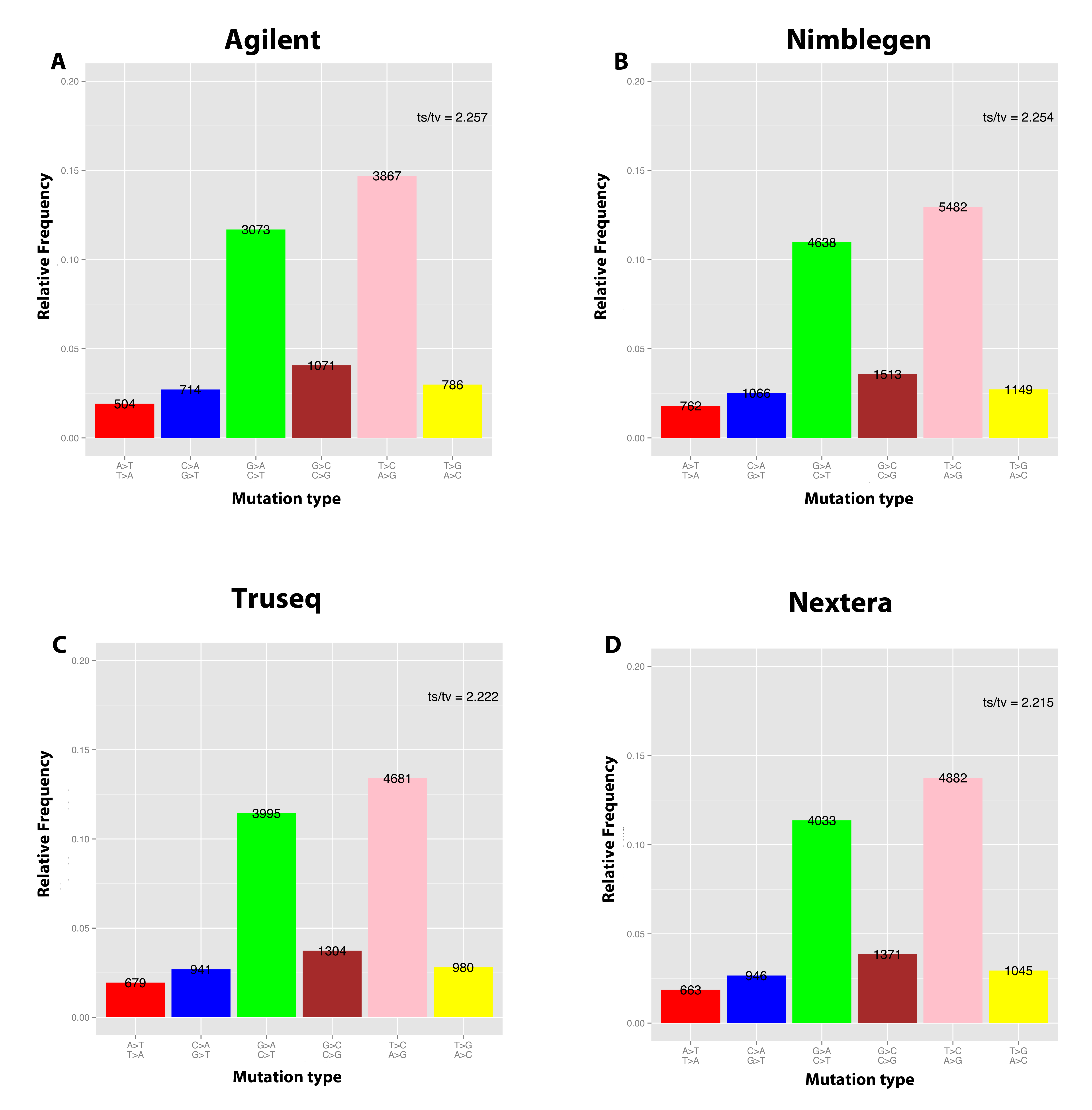

Supplement: Supplementary file 4 — Additional file 4: Figure S4: Mutation spectra by technology on intended target regions. Bar plots showing relative mutation frequency of different types of mutations for A) Agilent, B) NimbleGen, C) TruSeq, and D) Nextera technologies. Transition/Transversion (ts/tv) ratio indicated. (PNG 686 KB) [file 12864_2013_6212_MOESM4_ESM.png]

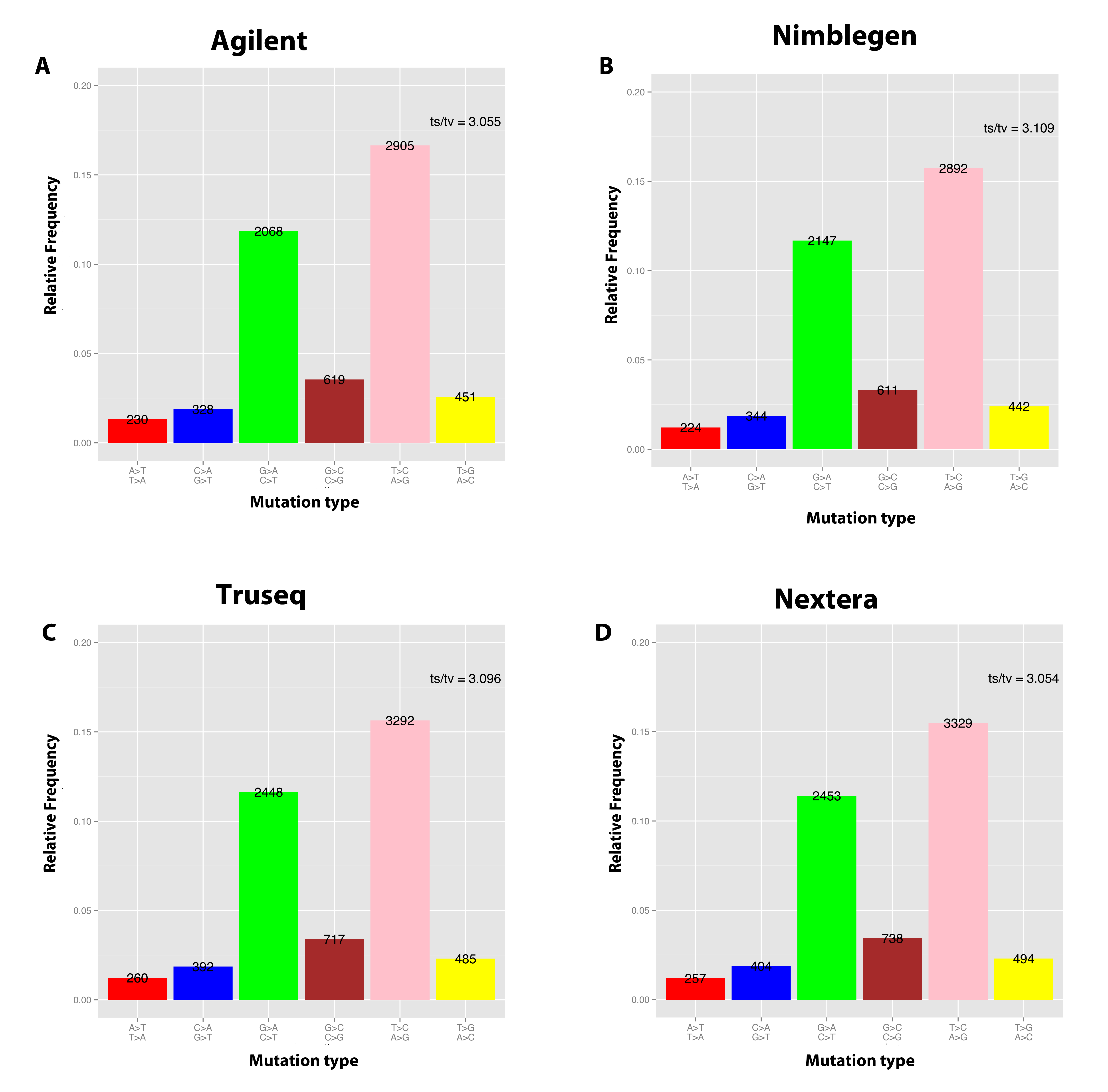

Supplement: Supplementary file 5 — Additional file 5: Figure S5: Mutation spectra from CCDS exonic regions by technology. Bar plots show the relative mutation frequency of different types of mutations for A) Agilent, B) NimbleGen, C) TruSeq, and D) Nextera technologies. Transition/Transversion (ts/tv) ratio indicated. (PNG 688 KB) [file 12864_2013_6212_MOESM5_ESM.png]

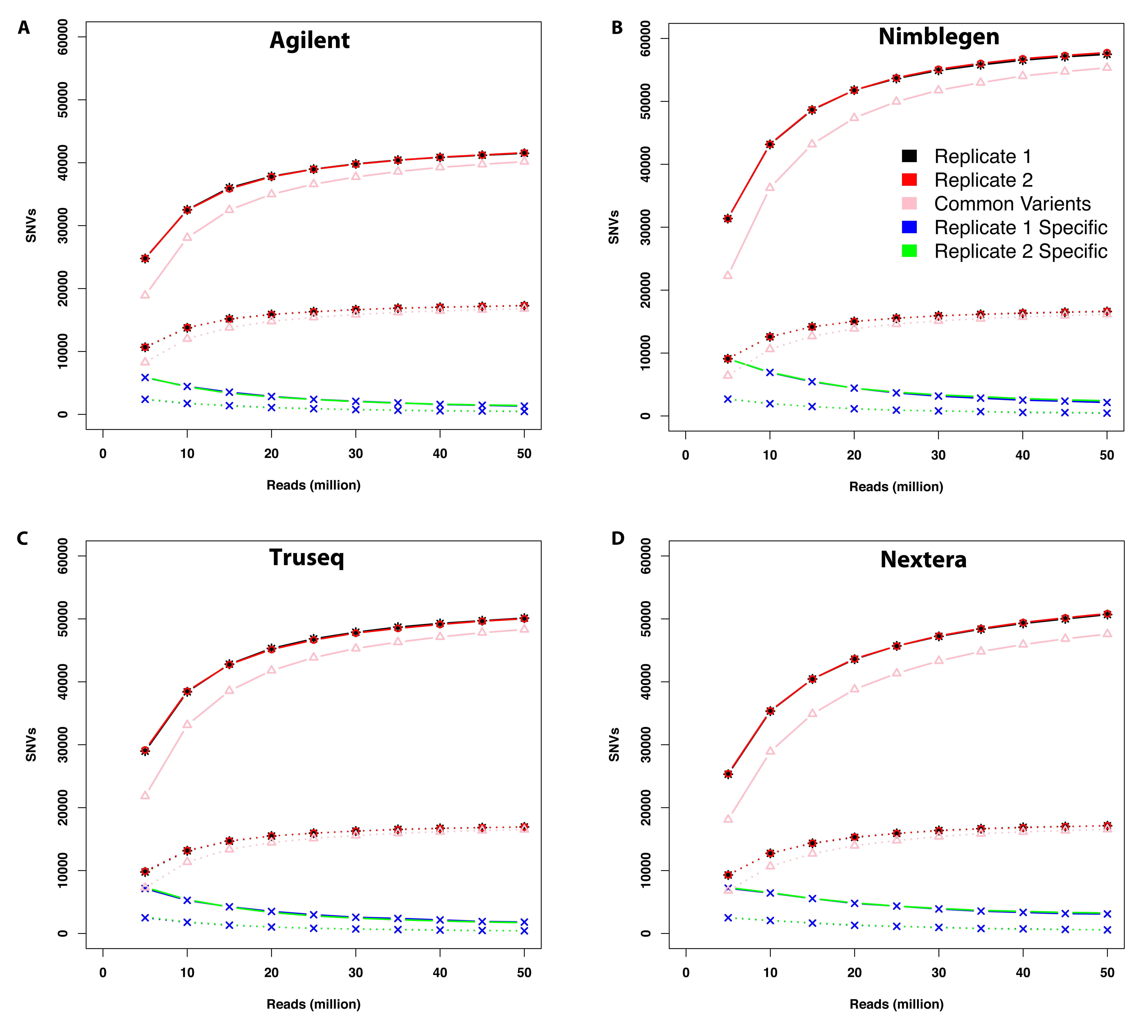

Supplement: Supplementary file 7 — Additional file 7: Figure S7: Comparison between two technical replicates in detecting SNVs, for A) Agilent, B) NimbleGen, C) TruSeq, and D) Nextera technologies. Smooth lines indicate SNVs detected on respective target regions, and dotted lines indicate SNVs detected on the target regions shared by all four technologies. Each figure shows the total number of SNVs detected by each replicate, common SNVs between replicates, and technology specific SNVs. (PNG 1 MB) [file 12864_2013_6212_MOESM7_ESM.png]

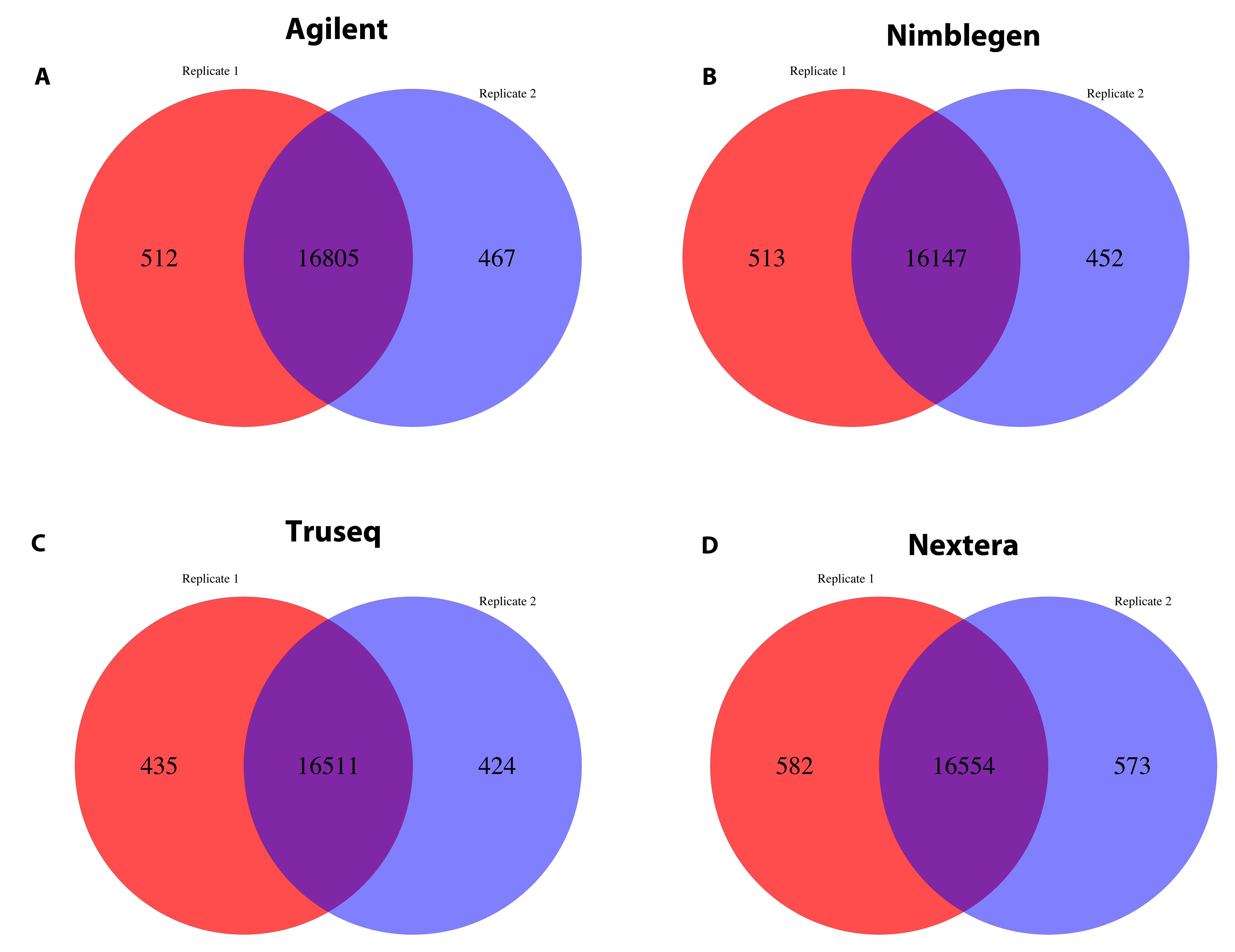

Supplement: Supplementary file 8 — Additional file 8: Figure S8: Comparison of SNVs detection by each technology at 50 million reads on regions shared by all four technologies between two replicates for A) Agilent, B) NimbleGen, C) TruSeq, and D) Nextera technologies. (PNG 585 KB) [file 12864_2013_6212_MOESM8_ESM.png]

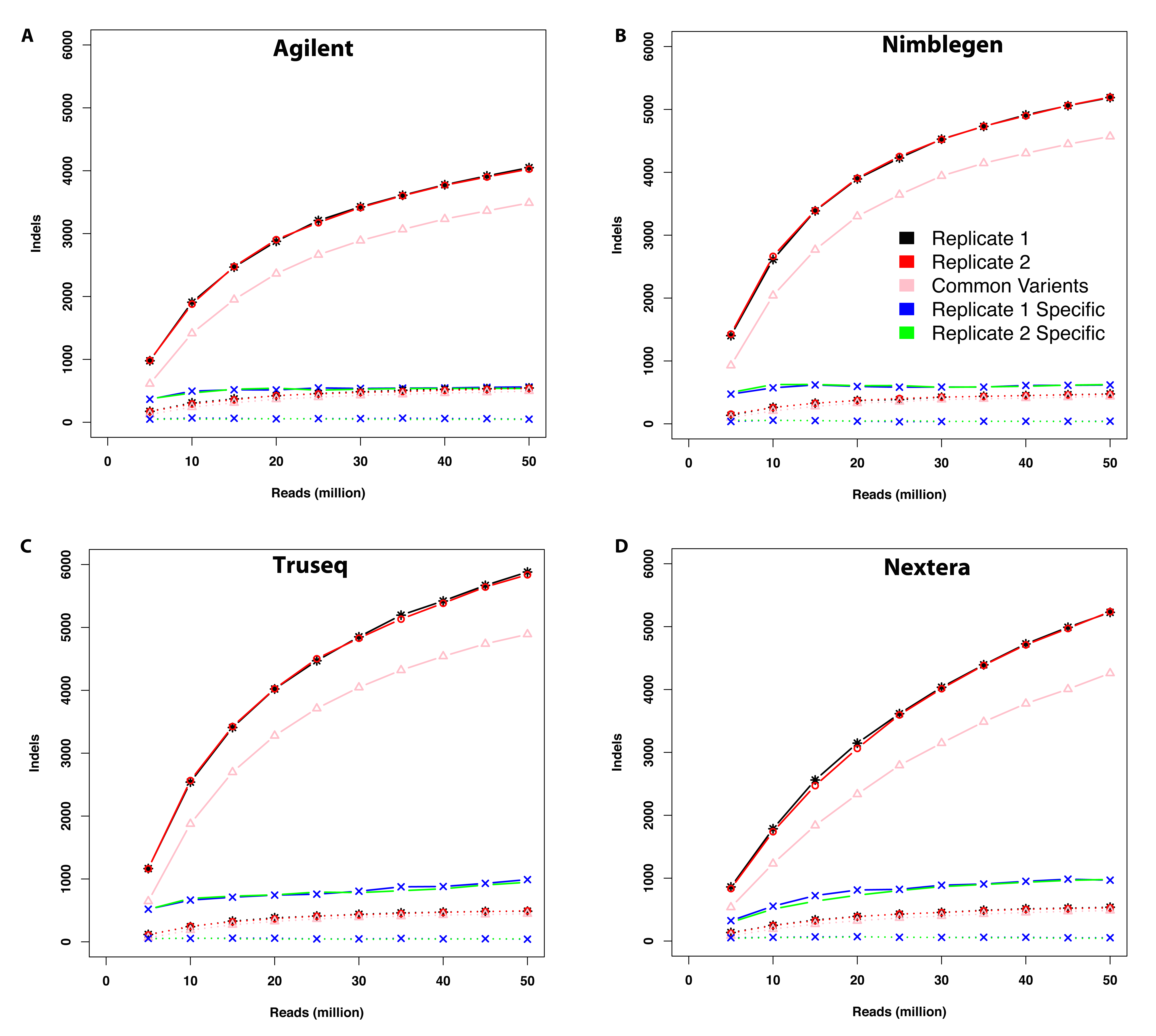

Supplement: Supplementary file 9 — Additional file 9: Figure S9: Comparison between two technical replicates in detecting indels, for A) Agilent, B) NimbleGen, C) TruSeq, and D) Nextera technologies. Smooth lines indicate indels detected on intended target regions, and dotted lines indicate indels detected on the target regions shared by all four technologies. Each figure shows the total number of indels detected by each replicate, common indels between the replicates, and technology specific indels. (PNG 1 MB) [file 12864_2013_6212_MOESM9_ESM.png]

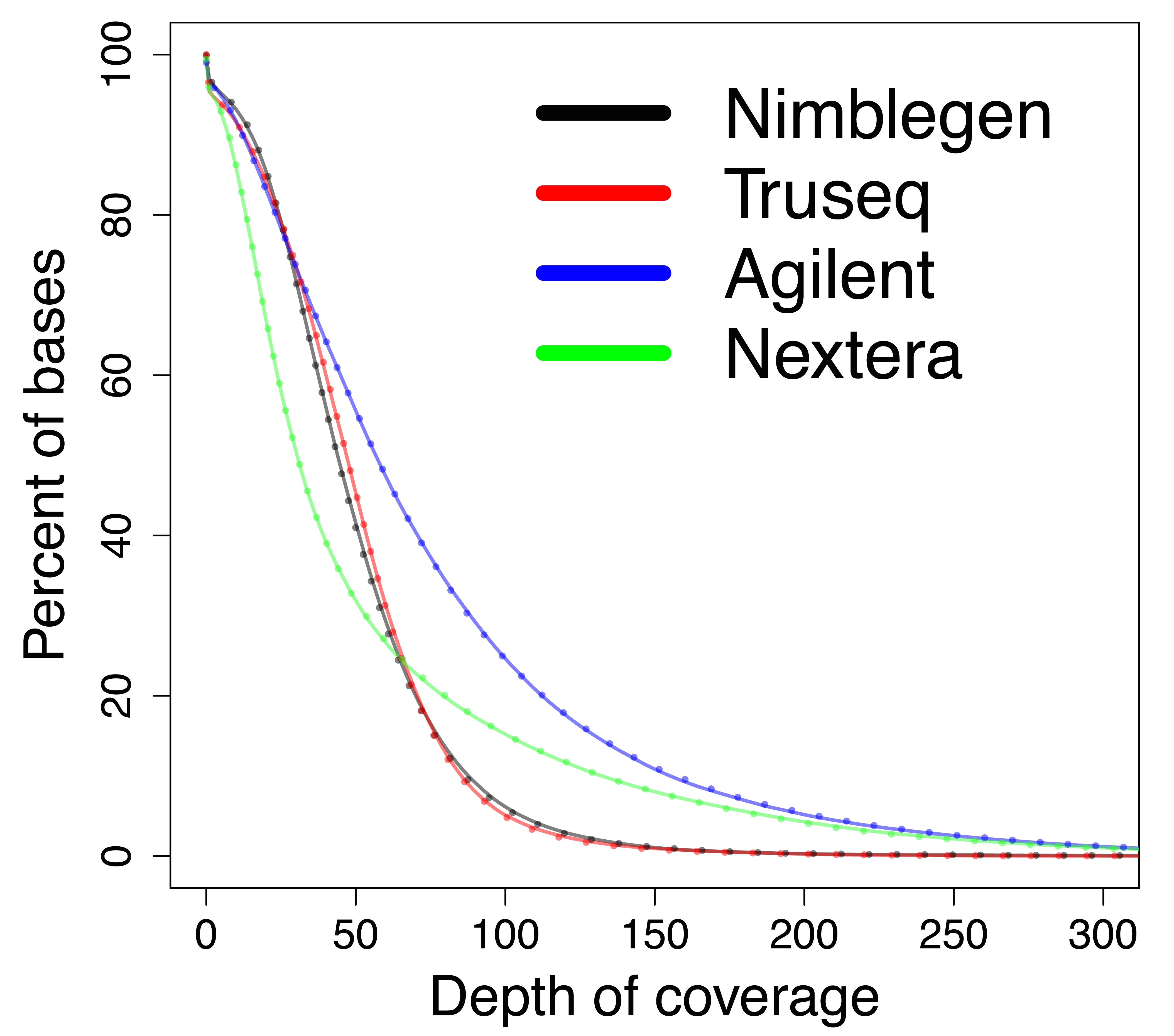

Supplement: Supplementary file 10 — Additional file 10: Figure S10: Coverage efficiency comparison by technology on cancer genes. The smooth line indicates replicate 1 and the dotted line indicates replicate 2. (PNG 703 KB) [file 12864_2013_6212_MOESM10_ESM.png]
